# The dual HDAC-PI3K inhibitor CUDC-907 displays single-agent activity and synergizes with PARP inhibitor olaparib in small cell lung cancer

**DOI:** 10.1186/s13046-020-01728-2

**Published:** 2020-10-17

**Authors:** Liying Ma, Xing Bian, Wenchu Lin

**Affiliations:** 1 High Magnetic Field Laboratory, Chinese Academy of Sciences, Hefei, 230031 Anhui P. R. China; 2 University of Science and Technology of China, Hefei, 230026 Anhui P. R. China; 3 Key Laboratory of High Magnetic Field and Ion Beam Physical Biology, Hefei Institutes of Physical Science, Chinese Academy of Sciences, Hefei, 230031 Anhui P. R. China

**Keywords:** SCLC, PI3K, HDAC, CUDC-907, PARP1, Olaparib, DSB repair, Combination treatment

## Abstract

**Background:**

Small cell lung cancer (SCLC) is a deadly neuroendocrine tumor with limited therapeutic options. Recent data suggest that histone deacetylases (HDACs) and the phosphatidylinositol 3-kinase (PI3K) pathway play essential roles in SCLC cell proliferation and survival.

**Methods:**

The inhibition of the PI3K signaling and HDAC activity by CUDC-907 was analyzed by western blotting. The effect of CUDC-907 on olaparib-induced DNA damage response was assessed by western blotting and Immunofluorescence staining. The cytotoxicity of CUDC-907 alone or in combination with olaparib in a panel of SCLC cell lines were evaluated by the CellTiter-Glo Luminescent Cell Viability Assay and flow cytometry. The in vivo effects of CUDC-907 and olaparib alone or in combination were examined using a patient-derived xenografts (PDX) model of SCLC.

**Results:**

CUDC-907 treatment downregulated MYC paralogs and FoxM1, induced G1 cell-cycle arrest, and impaired DNA double-strand break (DSB) repair capacity in SCLC cells, which produced a potent antiproliferative effect. Furthermore, we showed that CUDC-907 treatment enhanced the therapeutic efficacy of PARP inhibitor olaparib in SCLC cellular models and a PDX model. Mechanistic investigations demonstrated that CUDC-907 synergized with olaparib through the blockade of DSB repair pathways and downregulation of MYC paralogs and FoxM1.

**Conclusions:**

Our study uncovers that dual PI3K and HDAC inhibition by CUDC-907 exerts significant single-agent activity and strong synergistic effects with PARP inhibitor olaparib in SCLC, which thus provides a rational combination treatment strategy for SCLC clinical investigation.

## Background

Small cell lung cancer (SCLC), characterized by rapid tumor growth and early metastasis, represents one of the deadliest cancer types among all solid tumor malignancies [1, 2]. In the last couple of decades, the treatment landscape has not changed for SCLC. Nearly all patients eventually develop resistance to previously effective therapies, leading to a five-year survival rate at less than 7% [3]. Although considerable efforts have been invested and encouraging results have been obtained in preclinical studies, there were no therapeutic breakthroughs for this recalcitrant disease except the recent FDA approval of combined treatment using immunotherapy and chemotherapy [4]. Thus, developing effective targeted options is a pressing clinical need to improved therapeutic benefits for patients with SCLC.

A growing body of evidence indicates that both genetic changes and epigenetic alterations are extensively correlated with cancer initiation and progression [5]. In contrast to genetic mutations, the dynamic and reversible nature of epigenetic modifications makes epigenetic enzymes attractive to be harnessed for cancer therapy. Several epigenetic drugs such as histone deacetylase (HDAC) inhibitors showed promising anticancer activity and have been approved as anticancer agents by the FDA for hematological malignancies [6–9]. Previous studies have shown HDACi, as a single-agent or in combination with other small molecules, induces cell growth arrest, differentiation, and apoptosis in many cancer cell lines in vitro and in vivo, including SCLC [10–13].

Large-scale genomic and transcriptomic analyses have revealed that the PI3K/AKT/mTOR signaling pathway is frequently deregulated in SCLC [14–17], making it an attractive SCLC target. We and others have demonstrated that therapeutic targeting of this oncogenic pathway achieved encouraging anticancer activity in SCLC [17, 18]. However, acquired resistance to PI3K inhibitors is often developed due to concurrent activation of multiple oncogenic signaling networks. A potential strategy to overcome the resistance is to block the activation of those signaling pathways through histone deacetylase inhibition (HDACi). The combination of PI3K inhibitor with HDAC inhibitor has demonstrated promising anticancer effects in both preclinical and clinical studies in various cancers [19–22]. However, the combinatorial anticancer effect of the PI3K/AKT/mTOR pathway and HDAC inhibition has not been characterized in SCLC.

Poly ADP-ribose polymerase inhibitor (PARPi) has emerged as a promising anticancer agent in SCLC [23]. However, the efficacy of PARP inhibitors is limited by several mechanisms, including activation of homologous recombination (HR) and upregulation of the PI3K/AKT/mTOR pathway [24]. Thus, expanding the therapeutic potential of PARP inhibitors in SCLC is still an area of full interest. Herein, utilizing in vitro and in vivo models of SCLC, we explored the therapeutic merits of CUDC-907, a dual inhibitor of PI3K and HDAC. Furthermore, we set out to investigate whether CUDC-907 could improve the anticancer efficacy of PARP inhibitor olaparib through simultaneous, sustained disruption of the PI3K oncogenic pathway and DNA damage repair in SCLC.

## Materials and methods

### Cell cultures and reagents

Human small cell lung cancer (SCLC) cell lines were kept in RPMI-1640 containing 10% fetal bovine serum and 1% penicillin/streptomycin at 37 °C in a humidified incubator with 5% CO2. RPMI-1640 media, FBS, and penicillin/streptomycin were purchased from Gibco, Life Technologies (Carlsbad, CA, USA). CUDC-907 was purchased from MedChemExpress (Monmouth Junction, NJ, USA). Olaparib was obtained from Selleck Chemical (Shanghai, China). The compounds were first dissolved in DMSO and then diluted in medium (Sigma-Aldrich, Saint Louis, MO, USA).

### Cell viability assay

The small cell lung cancer cells were seeded into a 96-well dish in triplicates for each condition at an initial density of 3 × 10^3^ cells per well and cultured for 24 h. The following day the cells were treated with increasing concentrations of CUDC-907 or olaparib alone or in combination for 72 h. The cells were then harvested and analyzed using the CellTiter-Glo luminescent assay by following the manufacturer’s instructions. The IC50 values of CUDC-907 were calculated from the sigmoidal dose-response curve fits of data using the GraphPad Prism 7 software (GraphPad Software, Inc., La Jolla, CA, USA). Combination drug synergy was assessed using the CalcuSyn software (Biosoft) as previously described. In general, “CI < 1” denotes synergism, whereas “CI > 1” suggests antagonism [25].

### Cell cycle and apoptosis

SCLC cells were treated with 10 nM CUDC-907 or 10 μM olaparib alone or in combination for 24 h. After drug treatment, the cells were harvested and fixed by the dropwise addition of ice-cold EtOH. The fixed cells were stained with PI/RNase staining buffer (BD Biosciences, Franklin, NJ, USA), and then analyzed by a FACS Calibur (Sony Biotechnology, San Jose, CA, USA). The cell-cycle profiles were determined using ModFit software (Verity Software House, Topsham, ME, USA). To measure cell apoptosis, the cells were exposed to CUDC-907(10 nM) or olaparib (10 uM) alone or in combination for 48 h following which Annexin assays were conducted by incubating cells with Annexin V–fluorescein isothiocyanate and propidium iodide. Cell apoptosis was analyzed by a FACS Calibur (CytExpert, Beckman Coulter, Brea, CA, USA) and the data was analyzed using FlowJo V10 software (FlowJo LLC, Ashland, Oregon, USA).

### Western blotting and antibodies

Western blot samples were prepared as described previously [17]. Following treatment with 10 nM CUDC-907 or 10 μM olaparib alone or in combination for 24 h, the cells were lysed in RIPA buffer containing protease and phosphatase inhibitors. The extracted proteins were then separated using SDS-PAGE and transferred to PVDF membranes after quantification using a BCA Protein Assay Kit. Antibodies against the following proteins were used in this study: Phospho-Akt (Ser473, 1:1000,CST,#9271), Akt (1:1000,CST,#9712), Phospho-4E-BP1 (Ser65) (1:1000,CST, #9456),4E-BP1 (1:1000,CST,#9452), Phospho-S6 Ribosomal Protein (Ser235/236) (1:1000, CST, #4858), S6 Ribosomal Protein(1:1000, CST, #9202), acetyl-Histone H3(lys9, 1:1000, CST, #9649), PARP (1:1000, CST, #9542), Phospho-ChK1 (Ser317) (1:1000, CST, #12302), ChK1(1:1000, CST, #2360), p-DNA-PKcs (S2056) (Abcam, ab124918), DNA-PK(1:1000, Affinity, AF5340), c-MYC (1:1000, CST, #5605), MYCN (1:1000, CST, #9405), γ-H2AX (1:1000, CST, #9718), Rad51 (1:10000, Abcam, ab133534), 53BP1 (1:1000, CST, #88436), FoxM1 (1:1000, CST, #5436) and β-actin (1:10000, Transgen, HC201–02). Rabbit IgG (1:10,000, CST 7074) and mouse IgG (1:10,000, CST 7076) were used as secondary antibodies.

### Immunofluorescence microscopy

Immunofluorescence staining was performed as described previously [23]. Both suspended and adherent cells were fixed onto glass using 1% paraformaldehyde. After blocking and permeabilizing, the cells were incubated with anti-γH2AX (1:500, CST, #2577) and anti-Rad51 (1:500, Abcam, ab133534), followed by a fluorophore-conjugated secondary antibody for 1 h in a dark humidified chamber. After washing, the cells were counterstained using SlowFade Gold anti-fade reagent with DAPI (Invitrogen, Carlsbad, CA) and imaged using a Zeiss fluorescence microscope.

### Comet assay

Comet assay was performed as previously described [26]. Briefly, the cells were inoculated in a 6-well plate. After 48 h of CUDC-907(10 nM) or olaparib (10 μM) alone or combined treatment, the cells were collected and subjected to neutral single-cell gel electrophoresis. Following electrophoresis, the cells were stained with SYBR gold and viewed using a Zeiss fluorescence microscope.

### Quantitative real-time PCR

The total RNA from each sample was isolated with Trizol reagent (Thermo Scientific, Rockford, IL, USA) according to the manufacturer’s protocol. cDNA synthesis was synthesized by reverse transcription using cDNA Synthesis Kit (Roche, Mannheim, Germany). Q-PCR was performed using FastStart Essential DNA Green Master (vazyme, China) on a Roche LightCycler 96 Real-Time PCR System and the following primers:

**Table:** 

RAD51	Forward primer	5′-CAACCCATTTCACGGTTAGAGC-3’
	Reverse primer	5′-TTCTTTGGCGCATAGGCAACA-3’
MCM5	Forward primer	5′-ATGTCGGGATTCGACGATCCT-3’
	Reverse primer	5′-CCAGGTTGTAATGCCGCTTG-3’
CDC7	Forward primer	5′- GAGGCGTCTTTGGGGATTCAG-3’
	Reverse primer	5′-GGTCCTACTTGTAACTGTGCTG-3’
HK2	Forward primer	5′-GAGCCACCACTCACCCTACT-3’
	Reverse primer	5′-CCAGGCATTCGGCAATGTG-3’
LDHA	Forward primer	5′-ATGGCAACTCTAAAGGATCAGC-3’
	Reverse primer	5′-CCAACCCCAACAACTGTAATCT-3’
β-actin	Forward primer	5′-CATGTACGTTGCTATCCAGGC-3’
	Reverse primer	5′-CTCCTTAATGTCACGCACGAT-3’

### Patients-derived-xenograft (PDX) mouse model of SCLC

The use of SCLC specimens was approved by the Ethics Committee of Hefei Institutes of Physical Science, Chinese Academy of Sciences, in accordance with the Declaration of Helsinki. Fresh SCLC Primary tumor tissues were collected and transferred to a sterile petri dish containing Medium 199 plus penicillin/streptomycin. Then the tumor was cut into 2 × 2 × 2 mm blocks. The tumor blocks were implanted into the subcutaneous fat pad spaces of nonobese diabetic/severe combined immunodeficient mice that were monitored for the tumor to grow. For the drug treatment experiment, The 2 mm^3^ PDX tumor fragments were subcutaneously transplanted into the dorsal flanks of mice. When the tumor volume reached about 100mm^3^ in about two to 3 weeks, the mice were randomized into four groups and treated with DMSO control, CUDC-907, olaparib, and CUDC-907/olaparib combination for 15 days. For drug treatment, CUDC-907 was administrated to mice by oral gavage at a dose of 75 mg/kg/day. Olaparib was administrated to mice by intraperitoneal administration at a dose of 55 mg/kg/day. The tumor volume and weight of mice were measured every 3 days. Tumor sizes were measured using a caliper. Tumor weights were measured after 15 days of drug treatment. All mouse experiments were carried out according to a protocol approved by the Institutional Animal Care and Use Committee of Hefei Institutes of Physical Science. Female NOD/SCID (4–5-week old)) was purchased from Beijing Weitong Lihua Experimental Animal Limited Company.

### Histological and immunohistochemical analyses

The tumor tissues were fixed overnight with 4% formalin, followed by dehydration and paraffin embedding. Histopathological analysis was performed out on 4-μm sections stained with hematoxylin and eosin. Immunohistochemical analyses were carried out according to the immunohistochemical method described earlier [27]. Antibodies against the following proteins were used in this study: Ki67 (1:1000, CST #9449), Cleaved-Caspase 3 (1:300, CST, #9661), Rad51 (1:200, Abcam, ab133534) and γH2AX (Ser139) (1:500, CST, #2577), c-MYC (1:500, abcam, ab32072). Four to five random 40X fields were scored for each tumor sample. The staining intensity was quantified as the percentage of nuclear positive cells.

### Quantification and statistical analysis

The quantitative results were analyzed by double-tailed unpaired t-test using GraphPad Prism version 7.00 for Mac GraphPad Software. For in vitro analytical experiments, each test was repeated at least three times, with experiments being performed at least twice. Error bars for SD or SEM are shown. Where indicated in the figures, degrees of *p*-value significance are as follows: *p < 0.05, **p < 0.01, ***p < 0.001.

## Results

### Small cell lung cancer cells are highly sensitive to dual HDAC and PI3K inhibitor CUDC-907

**Fig. 1:**
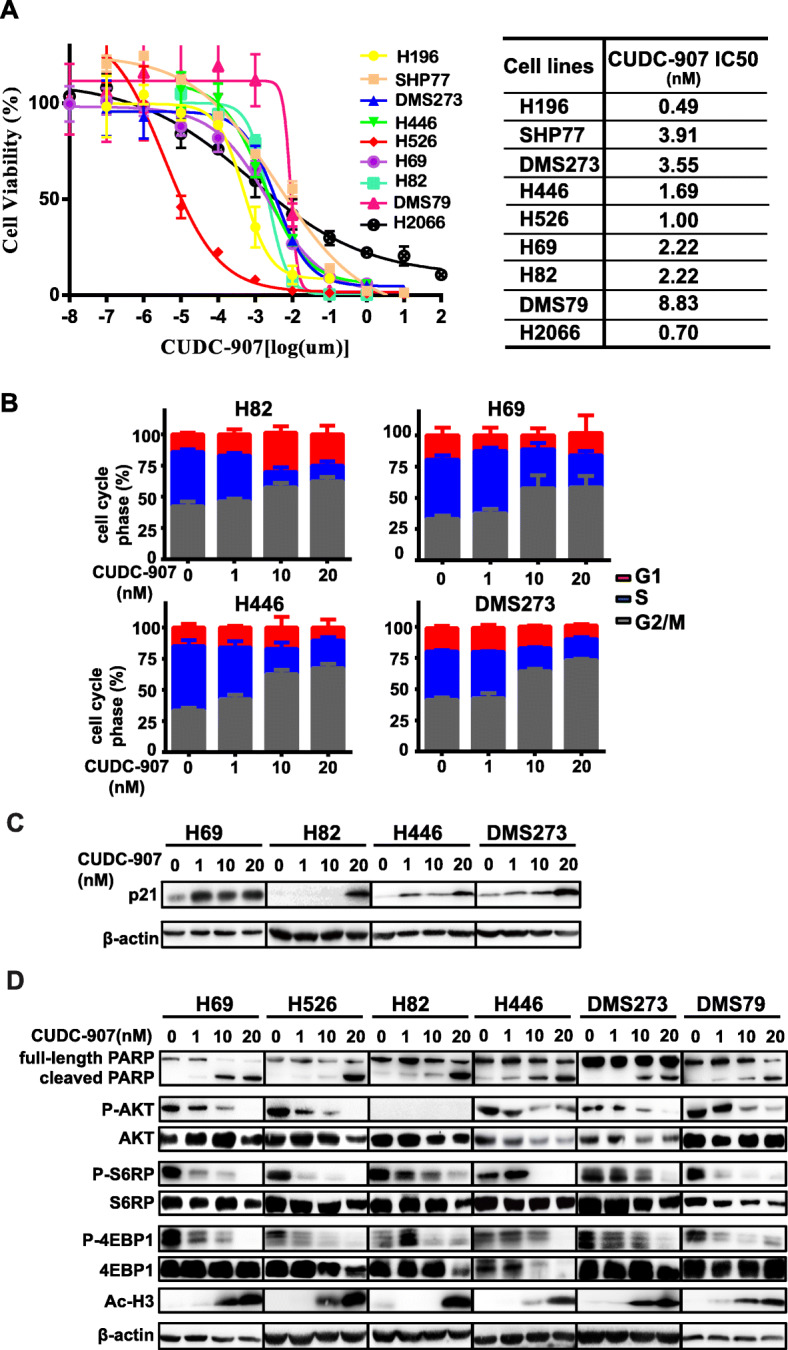
CUDC-907 induces cytotoxicity in a panel of SCLC cell lines. **a** Growth inhibition curves of CUDC-907 in a panel of SCLC cell lines. **b** Cell-cycle distribution of four SCLC cell lines following CUDC-907 treatment for 24 h by flow cytometry. **c**, **d** Western blot analysis of p21 (**c**), phospho-proteins downstream of PI3K and acetylated H3 (**d**) in SCLC cell lines upon treated with different concentrations of CUDC-907 for 24 h

As the PI3K signaling pathway and HDAC both are commonly dysregulated in SCLC cells, we wondered whether dual inhibition of phosphoinositide 3-kinase class I and pan histone deacetylase enzymes is effective in treating SCLC cells. A panel of SCLC cells was treated with increased concentrations of CUDC-907, a dual inhibitor of PI3K and HDAC, and the growth inhibitory effect of CUDC-907 was measured by CellTiter Glo assay after 3 days. We found that SCLC cells were highly sensitive to CUDC-907, with half-maximal inhibitory concentration (IC50) values at low nanomolar levels in all of SCLC cell lines examined (Fig. 1a). We then evaluated the effect of CUDC-907 on cell cycle progression by flow cytometry. CUDC-907 induced dose-dependent G1/S arrest in all the SCLC cell lines examined (Fig. 1b). Concordantly, p21, a key cell cycle regulator, was significantly induced by CUDC-907 (Fig. 1c). We also assessed the ability of CUDC-907 to induce apoptosis by western blotting. CUDC-907 treatment resulted in a dose-dependent accumulation of PARP cleavage in the cell lines tested (Fig. 1d). Together, these results suggest that CUDC-907 may exhibit potent cellular cytotoxicity against SCLC cells in part through cell cycle arrest and/or inducing cell apoptosis.

We then sought to investigate the mechanisms underlying the growth inhibitory effect of CUDC-907 as a single-agent in SCLC. We evaluated the changes induced by CUDC-907 in protein markers related to the PI3K pathways and HDAC. As expected, we observed that CUDC-907 inhibited the PI3K pathway, as indicated by the dose-dependent decreases in phosphorylation of AKT and its downstream targets, p-4EBP-1, and p-S6RP in all SCLC cell lines tested (Fig. 1d & Supplementary Fig. 1). Meanwhile, the acetylation histone 3 was robustly induced by CUDC-907 in a dose-dependent fashion, consistent with its effect as a histone deacetylase inhibitor (Fig. 1d). Together, we demonstrate that dual inhibition of PI3K and HDAC by CUDC-907 exhibits significantly enhanced anticancer activity against SCLC cells.

### CUDC-907 downregulates MYC signaling

**Fig. 2:**
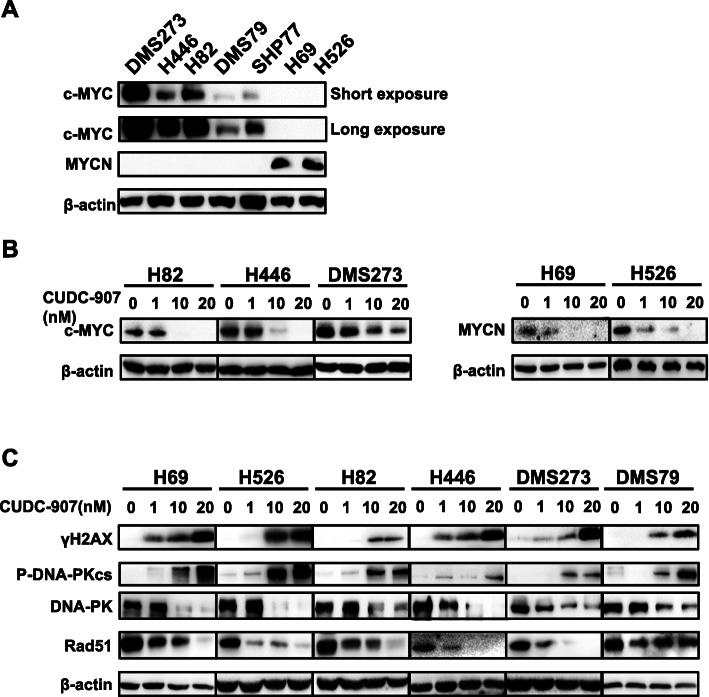
CUDC-907 results in dose-dependent downregulation of MYC paralogs and DDR pathway genes. **a** Western blot analysis of the basal expression of MYC paralogs in a panel of SCLC cell lines. **b**, **c** Western blot analysis of MYC paralogs in cells with amplification of MYC paralogs (**b**) and DDR pathway genes (**c**) in a panel of SCLC cell lines after treated with increased concentrations of CUDC-907 for 24 h

Previous studies showed that CUDC-907 and other HDAC inhibitors suppress MYC expression and inhibit MYC-dependent tumor proliferation in a variety of cancer types [20, 21, 28]. First, the expression of MYC paralogs was determined by western blot in a panel of SCLC cell lines. Western blot analysis showed that five cell lines (DMS273, H82, H526, H69, and H446) have a high expression level of c-MYC or MYCN and two cell lines (DMS79 and SHP77) have low or modest c-MYC expression, respectively (Fig. 2a). We next examined whether CUDC-907 treatment reduced the expression of Myc paralogs in SCLC. SCLC cell lines harboring either *c-MYC* or *MYCN* amplification were treated with increased concentrations of CUDC-907(Fig. 2b). Consistent with its potent effect in inhibiting the growth of cell lines with MYC alteration, western blot analysis showed that the abundance of c-MYC and MYCN were significantly reduced by CUDC-907 in a dose-dependent manner (Fig. 2b). Furthermore, the expression of MYC downstream targets, including *LDHA*, *HK2*, *MCM5*, and CDC7 was markedly reduced upon CUDC-907 or CUDC-907 combined with olaparib treatment in *MYC* paralog-amplified or overexpressed SCLC cells and tumors (Supplementary Figs. 2 & 5). Together, these data indicate that CUDC-907 effectively downregulates MYC signaling in SCLC, which might be partially attributable to the inhibitory effects of CUDC-907 in SCLC cell lines.

### Dual inhibition of PI3K and HDAC by CUDC-907 hampers DSB repair activity

Given the roles of HDAC and PI3K inhibitors in DNA damage repair, we evaluated the effect of CUDC-907 on DNA damage in SCLC cells. Western blot analysis revealed that treatment with CUDC-907 resulted in a dose-dependent increase of γH2AX, a marker of DNA double-strand breaks (DSBs) (Fig. 2c & Supplementary Fig. 4), indicating that CUDC-907 as monotherapy induced DNA damage in SCLC cells.

As CUDC-907 treatment induced dose-dependent accumulation of DNA DSB damage, we next sought to explore the effects of CUDC-907 on DSB repair pathways. CUDC-907 treatment resulted in significantly decreased Rad51 expression (Fig. 2c & Supplementary Fig. 3), reflecting compromised HR activity after CUDC-907 treatment. Furthermore, we wondered whether CUDC-907 treatment had an effect on the NHEJ repair pathway, another important DSB repair pathway that functions throughout the whole cell cycle. Interestingly, we found that CUDC-907 treatment resulted in significantly increased expression of phosphorylated DNA-PKcs at 2056 sites even though the expression of DNA-PKcs was markedly reduced (Fig. 2c & Supplementary Fig. 4), indicating decreased DNA end resection following CUDC-907 treatment. Together, our results indicate that CDUC-907 as a single-agent induces DNA damage and decreases DNA damage repair activity in SCLC cells.

### CUDC-907 potentiates the effect of PARP inhibitor olaparib in SCLC

**Fig. 3:**
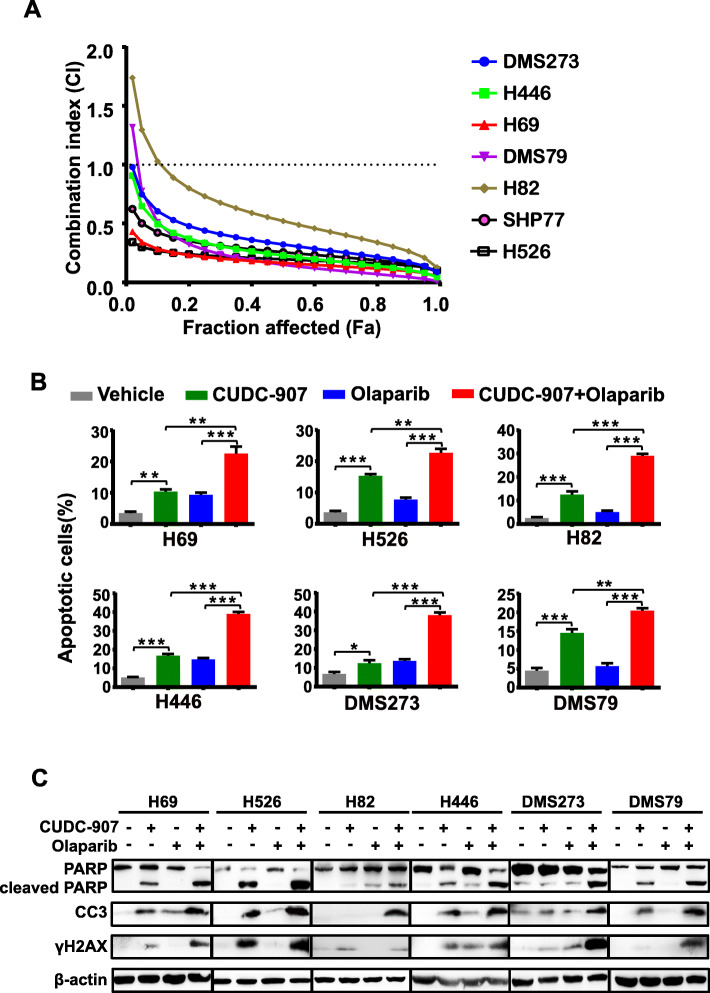
The combination effects of CUDC-907 and olaparib. **a** CellTiter-Glo Luminescent assays demonstrating the effects of 10 nM CUDC-907 and 10 μM olaparib in seven SCLC cell lines. **b** Apoptosis assessment of SCLC cells following 10 nM CUDC-907 and 10 μM olaparib as single-agents or in combination for 48 h. **c** Western blot analysis of cleavage of PARP and caspase 3, and γH2AX in SCLC cells upon treatment with 10 nM CUDC-907 or 10 μM olaparib alone or in combination for 24 h. CC3, cleaved caspase 3

PARP inhibitor, as a promising anticancer agent, has shown encouraging efficacy in SCLC. However, PARP inhibitor efficacy is limited by several factors, such as PI3K activity and HR status [24]. Based on the aforementioned finding, we hypothesized that CUDC-907 would synergize with olaparib to potentiate the anticancer effect of olaparib in SCLC cells. To test this possibility, we treated a panel of SCLC cell lines with increasing concentrations of CUDC-907 and olaparib as single-agents or in combination. The combined use of CUDC-907 and olaparib resulted in combination indexes (CI) of less than 1 in all of the concentrations tested in the SCLC cell lines examined (Fig. 3a), indicating a strong synergistic effect of CUDC-907 and olaparib on these SCLC cell lines.

To determine the effect of combined use of CUDC-907 and olaparib on cell apoptosis, apoptosis assay was conducted by Annexin V staining in SCLC cell lines. While CUDC-907 alone induced cell apoptosis to some extent, the apoptotic effect was further augmented by olaparib (Fig. 3b). In support of the results of flow cytometry, accumulation of PARP cleavage was observed in CUDC-907 treated cells, and to an even more significant extent, in cells treated with combined CUDC-907 and olaparib (Fig. 3c). Similarly, a substantial accumulation of caspase-3 cleavage was observed after combined treatment with CUDC-907 and olaparib (Fig. 3c). Together, these data indicate that CUDC-907 enhances cell apoptosis induced by olaparib and sensitize SCLC cells to olaparib.

### The combination of CUDC-907 and olaparib sufficiently enhances DNA damage

**Fig. 4:**
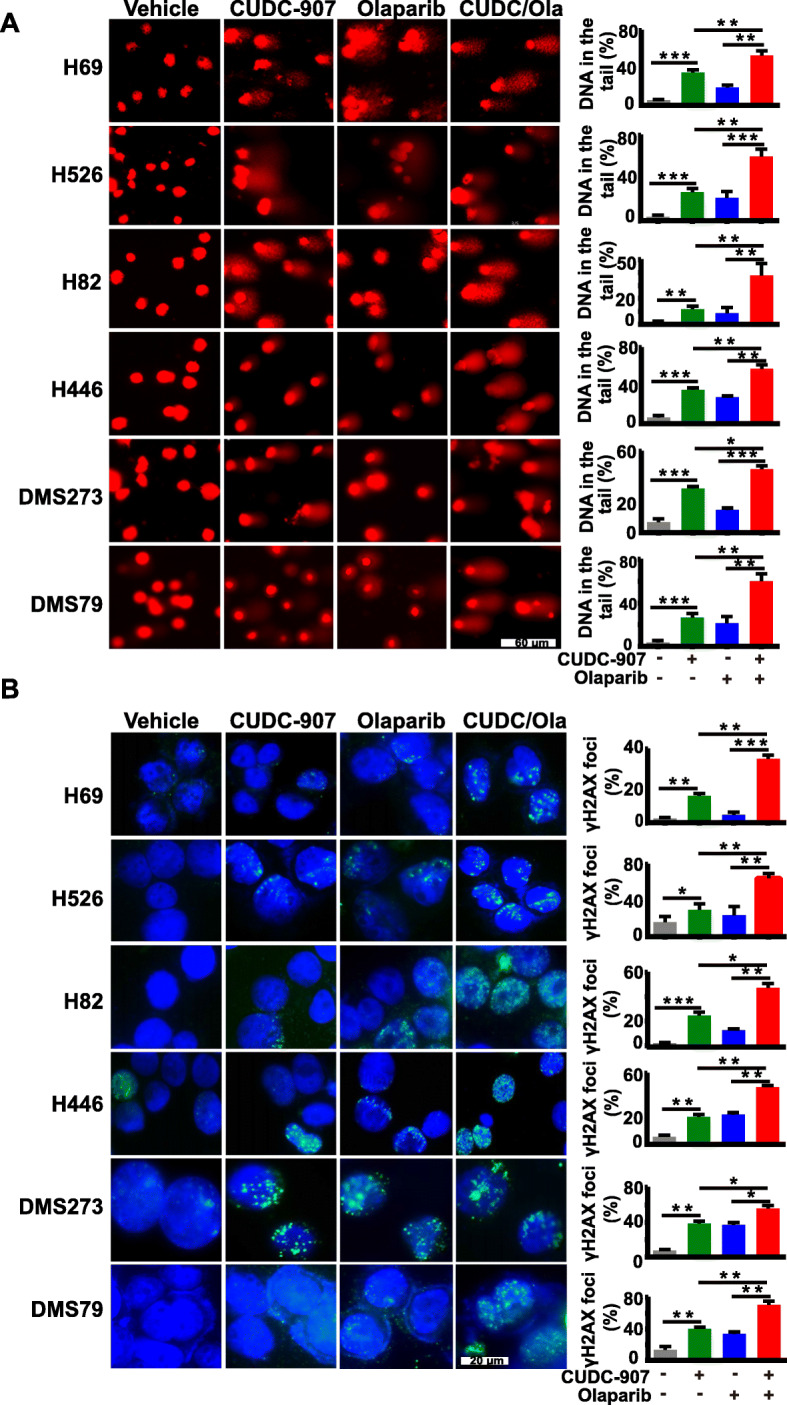
Effects of CUDC-907 and olaparib on DNA damage. **a** DNA damage detected by comet assay in SCLC cells upon treatment with 10 nM CUDC-907 or 10 μM olaparib alone and in combination for 48 h. Scale bar, 60 μm. DNA in the tail was used to measure DNA damage and assessed by CASP software (CaspLab). Quantification of the amount of DNA damage was presented mean ± S.D. **b** Representative images of immunofluorescent staining for γH2AX and quantification in SCLC cells treated with indicated drugs for 24 h, Scale bar, 20 μm

To dissect the synergistic anti-tumor mechanism of CUDC-907 and olaparib in SCLC cells, we evaluated the extent of DNA damage upon CUDC-907 as single-agent treatment and in combination with olaparib. CUDC-907 combined with olaparib significantly increased the expression levels of γH2AX than CUDC-907 alone (Fig. 3c). We then examined the extent of DNA damage using the comet assay. In the single-agent treatment arm, SCLC cells displayed DSBs to some degree in six SCLC cells examined, while the combination arm led to a remarkable accumulation of DNA in the tail, reflecting more severe DNA DSBs upon the combination treatment (Fig. 4a). In support of the comet assay results, immunofluorescence assay in six SCLC cell lines further confirmed much more elevated γH2AX foci in cells treated with the combination of CUDC-907 and olaparib than those treated with either drug alone (Fig. 4b). These results indicate that CUDC-907, combined with olaparib, leads to a synergistic effect on DNA damage in SCLC.

### Combining CUDC-907 and olaparib attenuates DNA damage repair

**Fig. 5:**
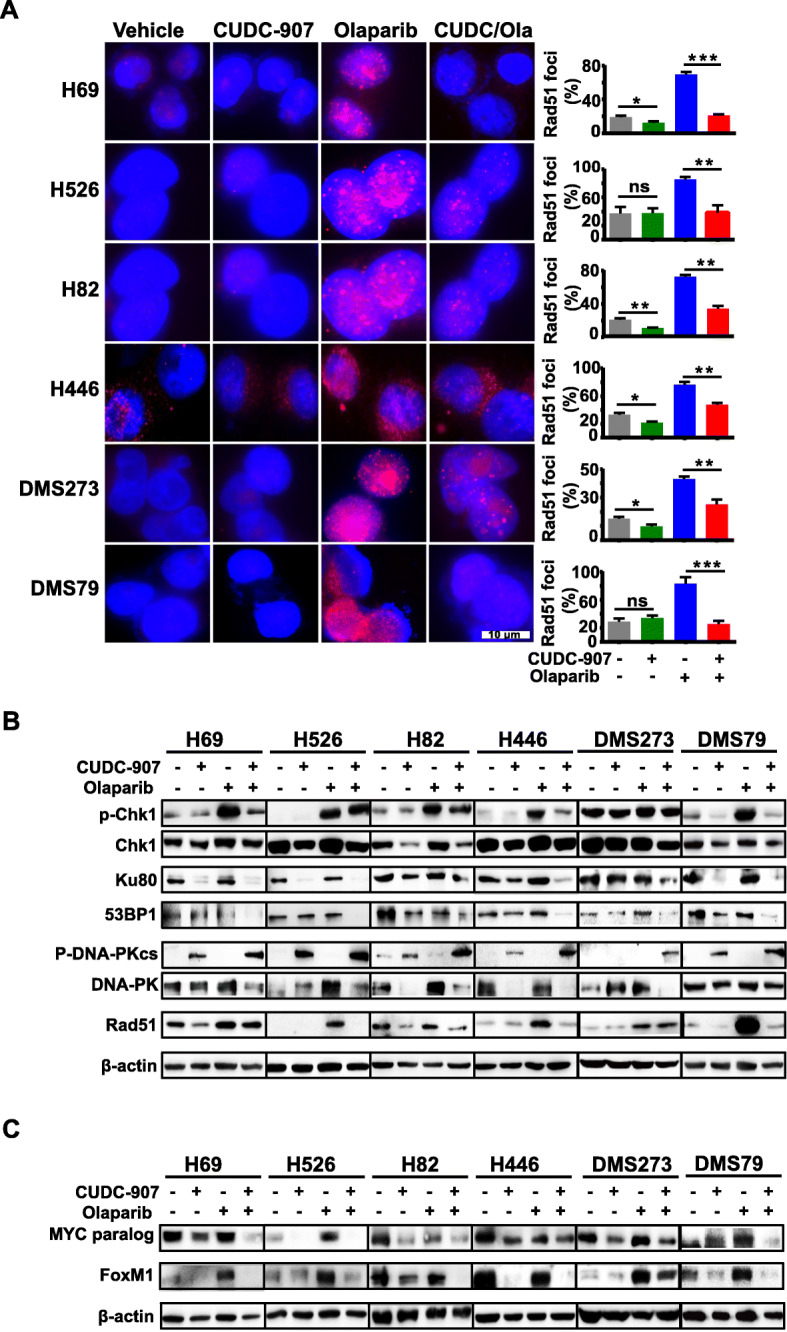
Effects of CUDC-907 and olaparib on the DNA repair pathways. **a** Representative images of Rad51 immunofluorescence staining in SCLC cells treated with drugs as indicated for 24 h. Scale bar, 10 μm. Quantification of Rad51 fluorescence intensities from three independent experiments was shown in right panels. **b**, **c** Western blot analysis of DDR proteins (**b**) and MYC paralogs and FoxM1 (**c**) in SCLC cells followed 10 nM CUDC-907 and 10 μM olaparib as single-agents or in combination treatment for 24 h. The antibody against MYCN was used to detect MYCN in H526 and H69 and anti-c-MYC was applied for the detection of c-MYC in rest of cell lines, β-actin was used as a loading control

To further discern the synergistic effect of CUDC-907 and olaparib on DNA damage in SCLC cells, we aimed to assess the impact of drug treatment on DNA damage repair activity. Immunofluorescence staining was performed to examine Rad51 foci formation. As expected, the formation of Rad51 nuclear foci was remarkably induced by olaparib, while CUDC-907 remarkably reduced olaparib-induced Rad51 foci in SCLC cell lines examined, suggesting impaired HR repair as a result of combined use of CUDC-907 and olaparib (Fig. 5a). To further explore whether CUDC-907 treatment led to impaired HR repair genes transcription, we conducted quantitative real-time PCR and western blot. We found that CUDC-907 significantly reduced the basal and olaparib-induced *Rad51* expression, indicating that CUDC-907 reduced Rad51 expression through inhibition of *RAD51* transcription (Fig. 5b and Supplementary Fig. 3). It is well documented that the ATR-ChK1 pathway is activated in response to DNA damage. Western blot analysis showed that olaparib substantially induced the p-ChK1 expression, which was counteracted by CUDC-907 in SCLC cells (Fig. 5b & Supplementary Fig. 6).

Nonhomologous end-joining (NHEJ) is another DSB repair pathway that requires 53BP1, Ku70/80 complex, and other c-NHEJ factors like DNA-PKcs in mammalian cells. To determine the effect of CUDC-907 treatment on NHEJ, SCLC cells were treated with CUDC-907 as a single-agent or in combination with olaparib for 24 h. Western blot analysis showed that CUDC-907 as a single-agent or in combination with olaparib significantly reduced 53BP1 expression (Fig. 5b), indicating decreased activity of DNA damage repair following CUDC-907 treatment. Evaluation of NHEJ revealed that SCLC cells treated with CUDC-907 alone or combined with olaparib showed significantly decreased Ku80 expression compared with the vehicle or olaparib-treated group (Fig. 5b). Furthermore, CUDC-907 as monotherapy or combined with olaparib remarkably enhanced p-DNA-PKcs (Fig. 5b & Supplementary Fig. 6).

Given that both MYC and FoxM1 regulate the genes that control DSB repair, we decided to check the expression of MYC paralogs and FoxM1 upon drug treatment. In accord with the observation shown in Fig. 2b, CUDC-907 treatment downregulated the expression of either c-MYC or MYCN (Fig. 5c). Interestingly, FoxM1 expression was markedly induced in several cell lines examined (H69, H526, DMS273, and DMS79) following olaparib treatment. The addition of CUDC-907 could downregulate FoxM1 expression induced by olaparib (Fig. 5c). Taken together, these results suggest that CDUC907 treatment resulted in function defects in HR and NHEJ, which might be due to the downregulation of MYC paralogs and FoxM1.

### The combined use of CUDC-907 and olaparib effectively suppresses tumor growth in a PDX model of SCLC

**Fig. 6:**
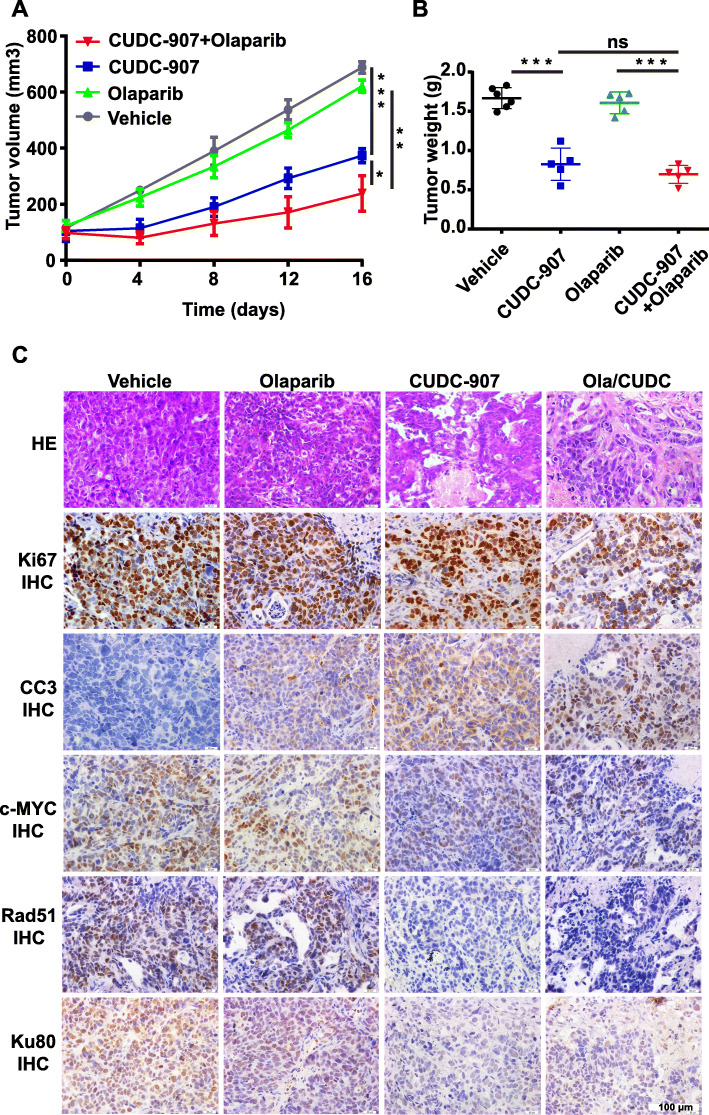
Therapeutic efficacy of CUDC-907 and olaparib as single-agents or in combination in vivo. **a**, **b)** Tumor volume curves (**a**) and tumor weights (**b**) of SCLC PDX tumors treated with CUDC-907, olaparib, or a combination of CUDC-907 and olaparib. **c** Representative immunohistochemistry images of Ki67, cleaved-caspase3 (CC3), c-MYC, Rad51, and Ku80 on PDX tumors treated with CUDC-907 and olaparib alone or in combination. Scale bar, 100 μm

We next evaluated the efficacy of CUDC-907 and olaparib as single-agents or in combination in SCLC *in* a PDX model of SCLC with MYC overexpression (Supplementary Fig. 7). No significant difference in body weight between control and treated mice was observed (data not shown), indicating that CUDC-907 monotherapy or in combination with olaparib is well tolerated. While olaparib as monotherapy showed limited efficacy, CUDC-907-treated mice exhibited more potent tumor growth inhibition than vehicle-treated mice. Remarkably, the combination of CUDC-907 and olaparib resulted in a more robust antitumor efficacy in tumor-bearing mice than either single-agent treatment group (Fig. 6a & b). To better understand the mechanism whereby CUDC-907 and olaparib cooperate to inhibit tumor growth, immunohistochemistry (IHC) analysis was performed on the tumor tissues treated with olaparib, CUDC-907 or a combination of these two drugs. Compared with single-agent treated groups, the combined use of CUDC-907 and olaparib caused substantially decreased Ki67 and increased cleaved-caspase3 staining positive cells (Fig. 6c). Concordantly, further IHC analysis of tumor tissues showed significantly reduced expression of HR repair protein Rad51 and c-NHEJ factor Ku80 in the combination group compared with single-agent alone (Fig. 6c), indicating accumulation of DNA double-strand breaks due to the attenuated DSB repair activity upon a CUDC-907 and olaparib combination. Moreover, the MYC expression was significantly reduced in CUDC-907 or the combination-treated arms (Fig. 6c) and the expression of MYC targets was markedly suppressed (Supplementary Fig. 5), which was consistent with the results observed in cell lines. Together, our data suggest that a combination of CUDC-907 and olaparib displays synergistic antitumor effects in a PDX model of SCLC.

## Discussion

Small cell lung cancer is a fatal neuroendocrine carcinoma with no effective targeted therapy. Despite the recent advance in the identification of novel therapeutic targets for SCLC, single-target drugs have been met with limitations. Therefore, multiple-target drugs might be an attractive avenue to increase cancer treatment effectiveness and sometimes decrease systemic toxicity. Although the dose of single-target HDAC or PI3K inhibitor can be adjusted to treat cancer, single-target drugs might cause serious adverse events or lead to resistance. Interestingly, the combination of these two inhibitors could generate a synergistic effect to inhibit tumor growth in vivo [20]. More importantly, CUDC-907 has been proved well tolerated. Therefore simultaneous inhibition of two pathways by CUDC-907 could be more effective than HDAC or PI3K inhibitor alone. The current study demonstrated that dual inhibition of PI3K and HDAC by CUDC-907 had potential anti-tumor efficacy in SCLC. Mechanistic investigations showed CUDC-907 induced cell cycle arrest and apoptosis, inhibited MYC and FoxM1, decreased DNA damage repair activity in SCLC cells. Most importantly, the study further showed that CUDC-907 potentiated the efficacy of PARP inhibitor olaparib in SCLC. The combined use of CUDC-907 and olaparib had a greater therapeutic efficacy against SCLC compared with either treatment alone.

The frequent activation of the PI3K/AKT/mTOR pathway has been demonstrated by several studies in both SCLC cell lines and clinical specimens [14–17]. Targeting the PI3K/AKT/mTOR signaling has shown potent treatment effects in SCLC cell lines. However, a preclinical study showed that PI3K inhibitor is effective in treating *PIK3CA* mutated, but not *PTEN*-deficient endometrial cells, thus limiting the efficacy of PI3K inhibitor as monotherapy [26]. Also, a previous clinical phase ‖ study indicated that mTOR inhibitor was not effective against SCLC [29]. Therefore, these studies demonstrated that identifying multiple-targeted drugs may expand treatment effects. CUDC-907, a dual inhibitor of PI3K and HDAC that targeting class Ɩ PI3Ks as well as class Ɩ and class ‖ HDAC has shown remarkable anti-tumor efficacy in multiple cancer types [20–22, 30]. However, the effect of CUDC-907 has not been investigated in SCLC. The current study demonstrated that CUDC-907 has strong antiproliferative effects in SCLC. Moreover, we showed that CUDC-907 strongly enhanced the effects of PARP inhibitor in SCLC. Our investigation provides a mechanistic demonstration for the efficacy of CUDC-907 single-agent activity and provides a rational combination modality for SCLC.

Transcriptomic analyses indicate that DNA damage repair genes are highly expressed in SCLC, limiting the efficacy of PARP inhibitors and other DNA-damaging agents. CUDC-907 showed intense activity to reduce the expression of crucial NHEJ and HR factors, including Rad51, Ku80, 53BP1, which provides the rationale to combine CUDC-907 with those DNA-damaging agents for the treatment of SCLC*.* A recent study showed that CUDC-907 reduces DDR-related gene expression through suppressing transcription factor FoxM1-mediated transcription [30]. The current study showed that CUDC-907 treatment significantly inhibits the expression of FoxM1 and MYC paralogs, two transcription factors frequently amplified or overexpressed in SCLC cells. Both FoxM1 and MYC paralogs can directly regulate the expression of DDR genes [30, 31]. Our data indicate that CUDC-907 might decrease DDR gene expression through the downregulation of FoxM1 and MYC paralogs.

*MYC* paralogs are exclusively amplified or overexpressed in SCLC, leading to treatment resistance. We showed that CUDC-907 treatment resulted in a dose-dependent downregulation of *MYC* paralogs in a panel of SCLC cell lines. Furthermore, our results also showed that the downstream targets of MYC signaling were inhibited by CUDC-907 treatment. Therefore, MYC itself and its downstream signaling besides DDR pathways at least partially contribute to the antitumor effects of CUDC-907.

PARP inhibitors represent the most anticipated anti-tumor drugs for SCLC [23]. The current study indicated that CUDC-907 treatment led to decreased HR factor Rad51 and NHEJ factor Ku80 and 53BP1, indicating that targeting DSB repair pathways with dual HDAC-PI3K inhibitors is a promising strategy to improve PARPi efficacy in SCLC. Beyond the existing mechanism of CUDC-907 that present in this manuscript, more mechanistic investigations are needed to support the potential utility of this combination in the treatment of SCLC. Nonetheless, this study demonstrates the single-agent activity of CUDC-907 in SCLC, and most importantly, establishes the therapeutic rationale for the combination of dual HDAC-PI3K inhibitors and olaparib in SCLC. Our study may provide a new combination strategy for further SCLC clinical investigation.

## Conclusion

The current study demonstrated that dual inhibition of PI3K and HDAC by CUDC-907 had potential anti-tumor efficacy in SCLC. Mechanistic investigations showed CUDC-907 induced cell cycle arrest and apoptosis, inhibited MYC and FoxM1, decreased DNA damage repair activity in SCLC cells. Most importantly, the study further showed that CUDC-907 potentiated the efficacy of PARP inhibitor olaparib in SCLC. The combined use of CUDC-907 and olaparib had a greater therapeutic efficacy against SCLC than either treatment alone. The current study indicated that CUDC-907 treatment led to decreased HR factor Rad51 and NHEJ factor Ku80 and 53BP1, indicating that targeting DSB repair pathways with dual HDAC-PI3K inhibitors is a promising strategy to improve PARPi efficacy in SCLC.

## Supplementary information

**Additional file 1: Supplementary Figure 1.** Relative amount of phosphorylated proteins were determined by densitometric analysis.The quantification data presented were the average densitometric value of three independent western blotting experiments. One of three experiments with similar results is shown in Fig. 1d.

**Additional file 2: Supplementary Figure 2.** Effects of CUDC-907 and olaparib on the expression of MYC targets in SCLC cells. RT-qPCR analysis of the expression of MYC targets in SCLC cells treated as indicated drugs for 24 h. Gene expression was normalized to *β-actin*. Error bars represent mean ± S.D. *P < 0.05; **P < 0.01; ***P < 0.001.

**Additional file 3: Supplementary Figure 3.** Effects of CUDC-907 and olaparib on the expression of *RAD51* in SCLC cells. RT-qPCR analysis of *RAD51* expression in SCLC cells treated with 10 nM CUDC-907 and 10 μM olaparib alone or in combination for 24 h. Gene expression was normalized to *β-actin*. Error bars represent mean ± S.D. *P < 0.05; **P < 0.01; ***P < 0.001.

**Additional file 4: Supplementary Figure 4.** Relative amount of DDR proteins were determined by densitometric analysis. The quantification data presented were the average densitometric value of three independent western blotting experiments. One of three experiments with similar results is shown in Fig. 2c.

**Additional file 5: Supplementary Figure 5.** Effects of CUDC-907 and olaparib on the expression of MYC targets in vivo. RT-qPCR analysis of the expression of MYC targets in PDX tissues treated as indicated drugs for 15 days. Gene expression was normalized to *β-actin*. Error bars represent mean ± S.D. *P < 0.05; **P < 0.01; ***P < 0.001.

**Additional file 6: Supplementary Figure 6.** Relative amount of DDR proteins were determined by densitometric analysis. The quantification data presented were the average densitometric value of three independent western blotting experiments. One of three experiments with similar results is shown in Fig. 5b.

**Additional file 7: Supplementary Figure 7.** Representative images of H & E and Immunohistochemical staining for c-MYC (1:500, abcam, ab32072) in SCLC primary tumors and PDX specimens.

## Data Availability

All data generated or analyzed during this study are included in this published article.

## Abbreviations

SCLC: Small cell lung cancer
HDAC: Histone deacetylase
PI-3 K: Phosphatidylinositol 3 kinase
PDX: Patient-derived xenografts
SCLC: Small cell lung cancer
DSB: DNA double-strand break
HR: Homologous recombination;
PARPi: Poly ADP-ribose polymerase inhibitor
NHEJ: Nonhomologous end-joining
Rad51: Rad51 recombinase
IC50: Inhibitory concentration
p-AKT: Phosphorylated-AKT
p-DNA-PK: Phosphorylated-DNA-dependent protein kinase
PARP: Poly ADP-ribose polymerase
IHC: Immunohistochemistry
FoxM1: Forkhead box M1
