# Correction to: The dual HDAC-PI3K inhibitor CUDC-907 displays single-agent activity and synergizes with PARP inhibitor olaparib in small cell lung cancer

**DOI:** 10.1186/s13046-020-01805-6

**Published:** 2021-01-05

**Authors:** Liying Ma, Xing Bian, Wenchu Lin

**Affiliations:** 1 High Magnetic Field Laboratory, Chinese Academy of Sciences, Hefei, 230031 Anhui P. R. China; 2 University of Science and Technology of China, Hefei, 230026 Anhui P. R. China; 3 Key Laboratory of High Magnetic Field and Ion Beam Physical Biology, Hefei Institutes of Physical Science, Chinese Academy of Sciences, Hefei, 230031 Anhui P. R. China

**Correction to: J Exp Clin Cancer Res (2020) 39:219**

**https://doi.org/10.1186/s13046-020-01728-2**

Following the publication of the original article [1], it was noted that the representative images of Rad51 immunofluorescence staining of H82 cell line in the original Figure 5A of this article were presented incorrectly. The errors occurred because the representative images of H526 were not appropriately labeled for H82 by error during the figure preparation. The updated Figure 5A was generated and is shown below based on the original raw data. The authors declare that these corrections do not change the results or conclusions of this paper. The authors apologize for this error.

The original article has been corrected.


