# Supplementary figures and images for: The dual HDAC-PI3K inhibitor CUDC-907 displays single-agent activity and synergizes with PARP inhibitor olaparib in small cell lung cancer
Source: J Exp Clin Cancer Res. 2020 Oct 17;39:219. doi: 10.1186/s13046-020-01728-2 (PMC7568419; doi:10.1186/s13046-020-01728-2)

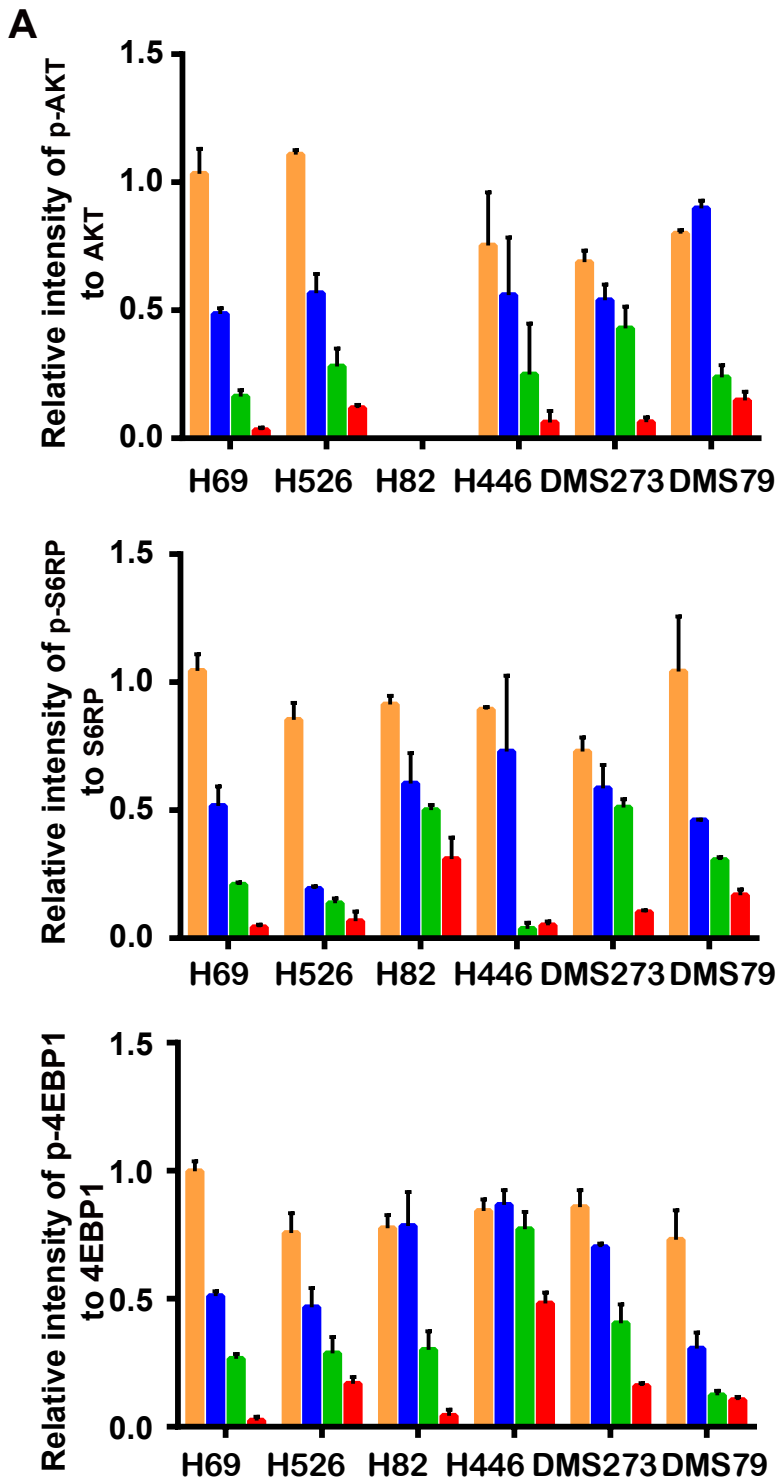

**Figure S1.**

Supplement: Supplementary file 1 — Additional file 1: Supplementary Figure 1. Relative amount of phosphorylated proteins were determined by densitometric analysis.The quantification data presented were the average densitometric value of three independent western blotting experiments. One of three experiments with similar results is shown in Fig. 1d. [file 13046_2020_1728_MOESM1_ESM.pdf]

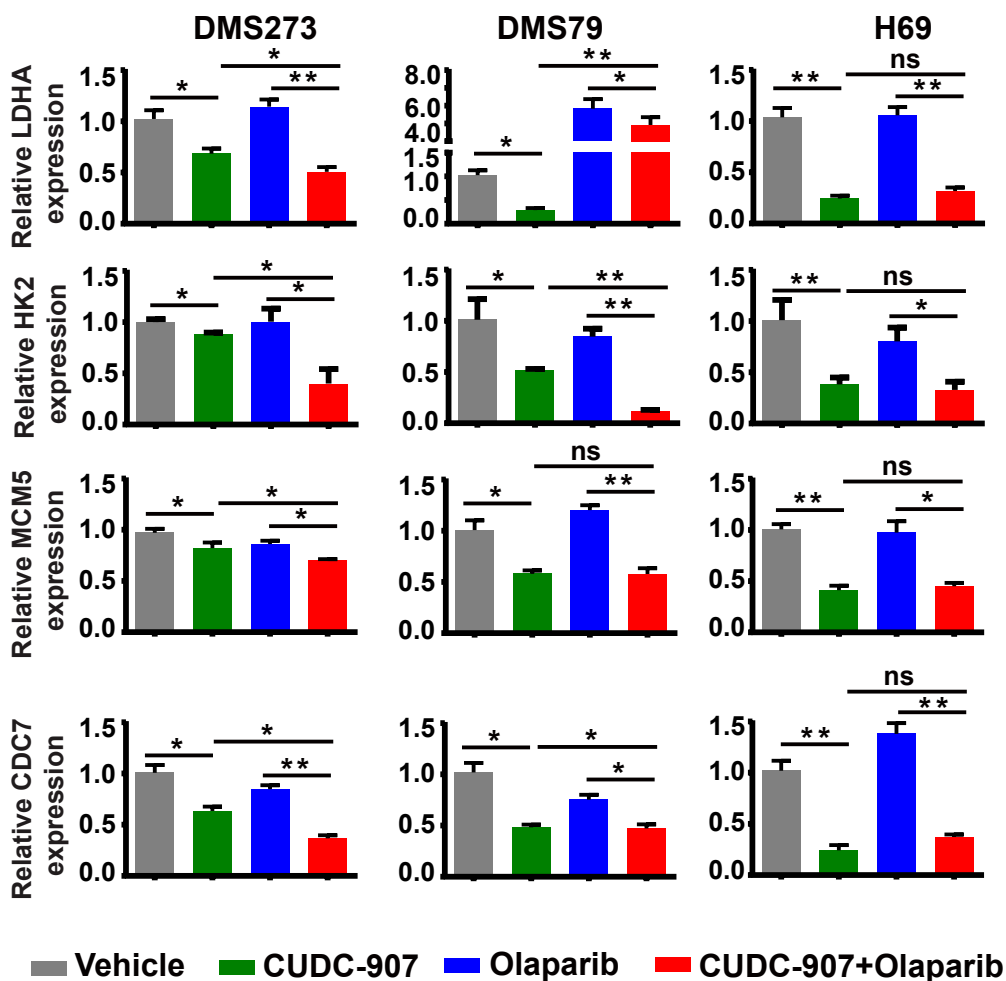

**Figure S2.**

Supplement: Supplementary file 2 — Additional file 2: Supplementary Figure 2. Effects of CUDC-907 and olaparib on the expression of MYC targets in SCLC cells. RT-qPCR analysis of the expression of MYC targets in SCLC cells treated as indicated drugs for 24 h. Gene expression was normalized to β-actin. Error bars represent mean ± S.D. *P < 0.05; **P < 0.01; ***P < 0.001. [file 13046_2020_1728_MOESM2_ESM.pdf]

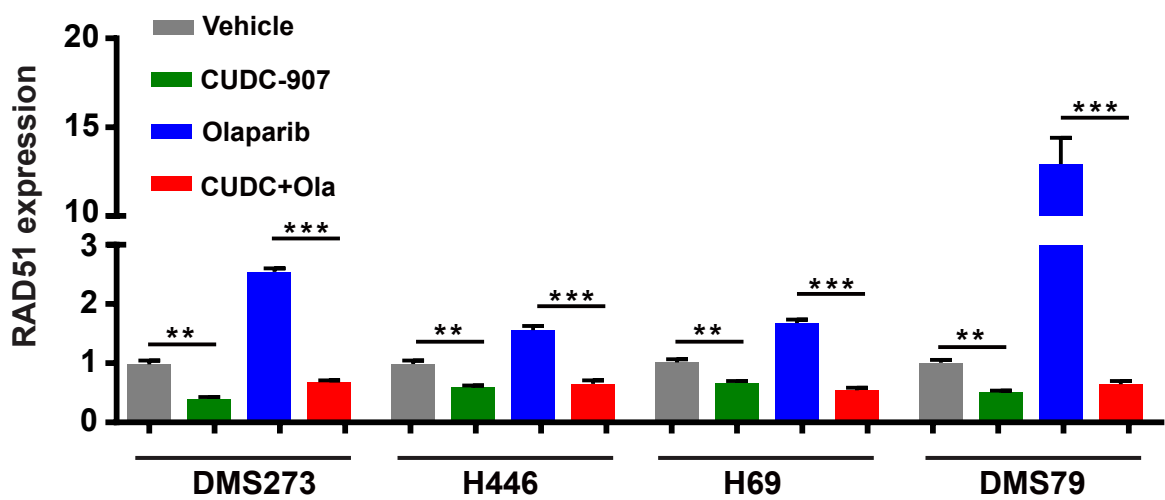

Figure S3.

Supplement: Supplementary file 3 — Additional file 3: Supplementary Figure 3. Effects of CUDC-907 and olaparib on the expression of RAD51 in SCLC cells. RT-qPCR analysis of RAD51 expression in SCLC cells treated with 10 nM CUDC-907 and 10 μM olaparib alone or in combination for 24 h. Gene expression was normalized to β-actin. Error bars represent mean ± S.D. *P < 0.05; **P < 0.01; ***P < 0.001. [file 13046_2020_1728_MOESM3_ESM.pdf]

**A**

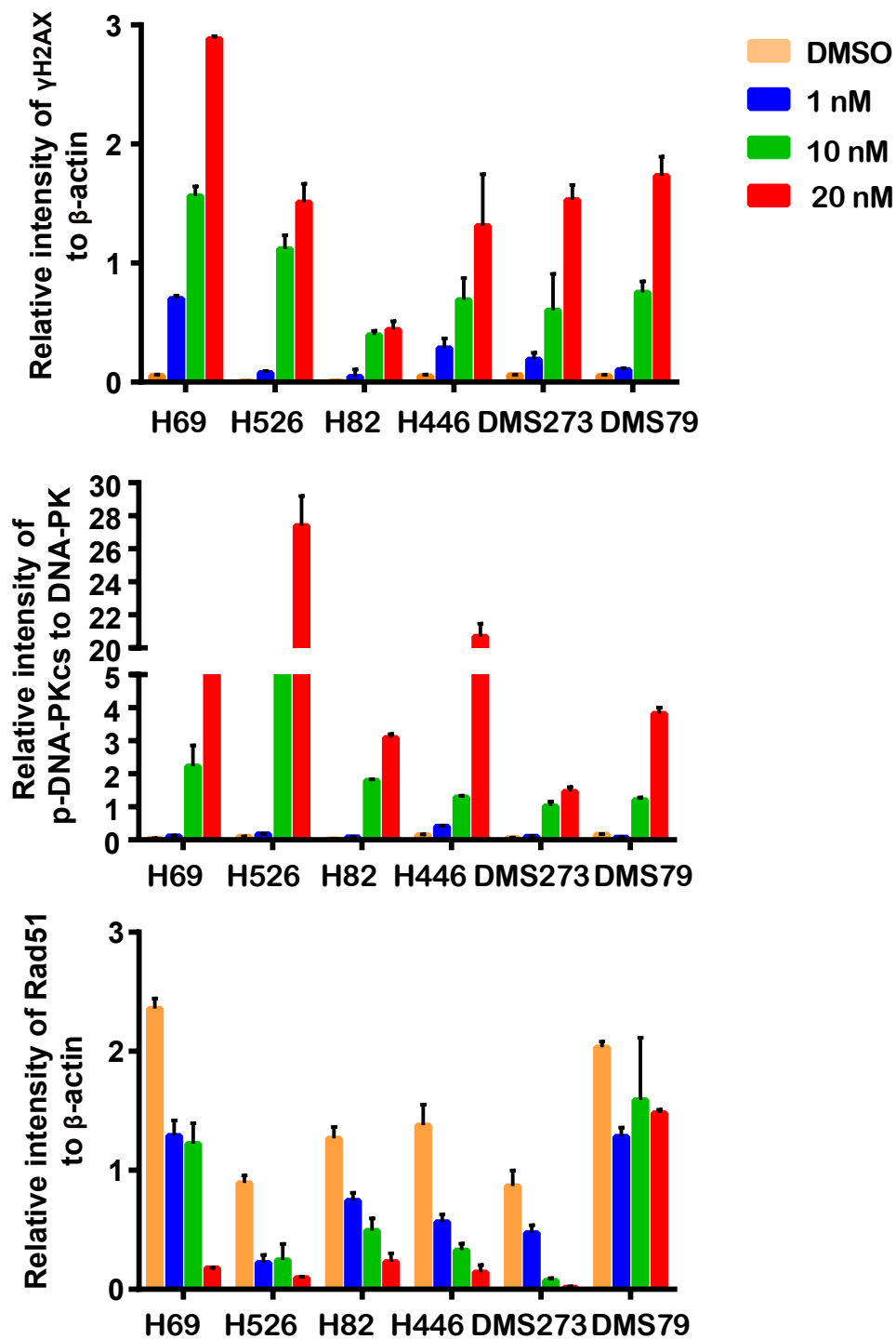

**Figure S4.**

Supplement: Supplementary file 4 — Additional file 4: Supplementary Figure 4. Relative amount of DDR proteins were determined by densitometric analysis. The quantification data presented were the average densitometric value of three independent western blotting experiments. One of three experiments with similar results is shown in Fig. 2c. [file 13046_2020_1728_MOESM4_ESM.pdf]

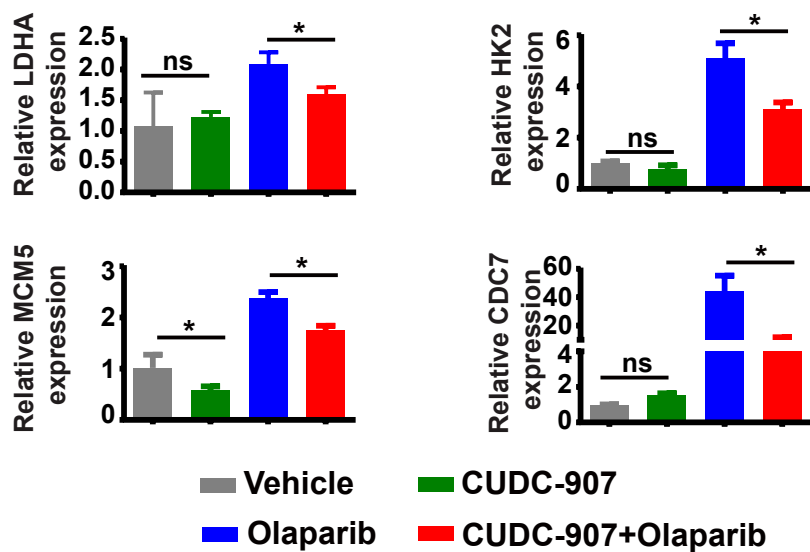

**Figure S5.**

Supplement: Supplementary file 5 — Additional file 5: Supplementary Figure 5. Effects of CUDC-907 and olaparib on the expression of MYC targets in vivo. RT-qPCR analysis of the expression of MYC targets in PDX tissues treated as indicated drugs for 15 days. Gene expression was normalized to β-actin. Error bars represent mean ± S.D. *P < 0.05; **P < 0.01; ***P < 0.001. [file 13046_2020_1728_MOESM5_ESM.pdf]

**A**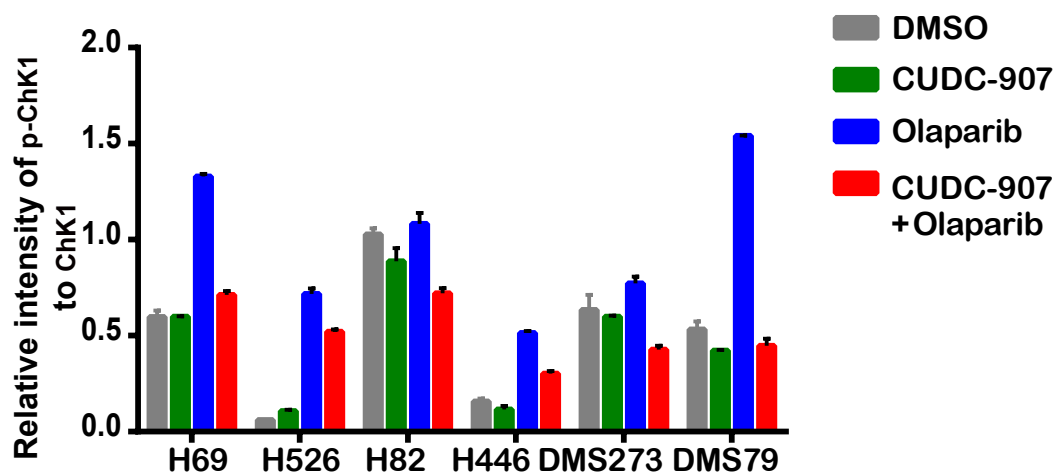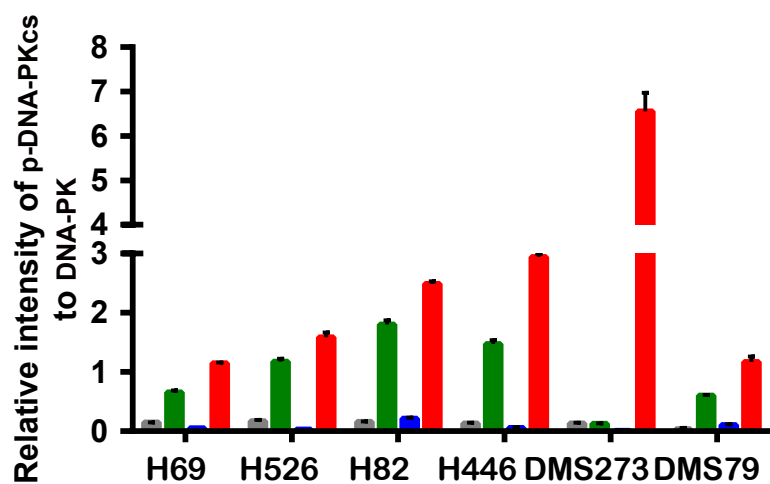

**Figure S6.**

Supplement: Supplementary file 6 — Additional file 6: Supplementary Figure 6. Relative amount of DDR proteins were determined by densitometric analysis. The quantification data presented were the average densitometric value of three independent western blotting experiments. One of three experiments with similar results is shown in Fig. 5b. [file 13046_2020_1728_MOESM6_ESM.pdf]

**A**

**Primary tumor**

**PDX secimen**

**H & E**

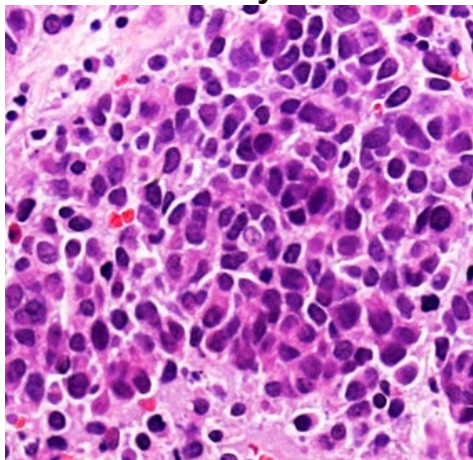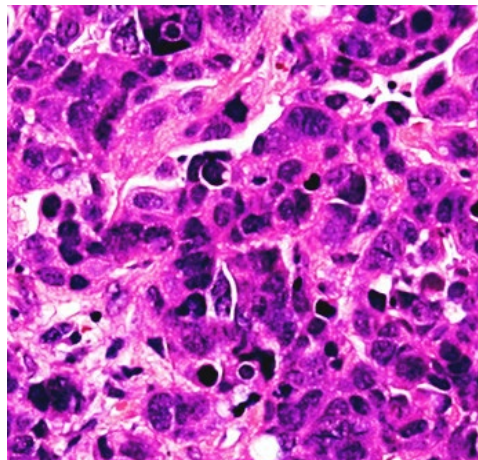

**c-MYC  
IHC**

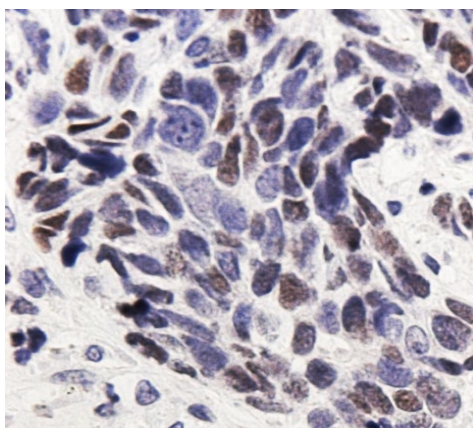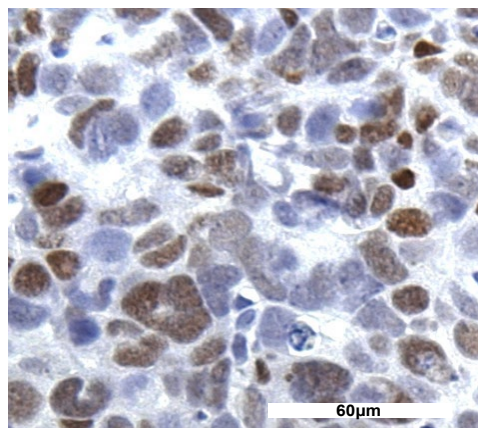

**Figure S7.**

Supplement: Supplementary file 7 — Additional file 7: Supplementary Figure 7. Representative images of H & E and Immunohistochemical staining for c-MYC (1:500, abcam, ab32072) in SCLC primary tumors and PDX specimens. [file 13046_2020_1728_MOESM7_ESM.pdf]
